# Exciplex-forming cohost systems with 2,7-dicyanofluorene acceptors for high efficiency red and deep-red OLEDs

**DOI:** 10.1038/s41598-024-52680-6

**Published:** 2024-01-30

**Authors:** Yi-Sheng Chen, I-Hung Lin, Hsin-Yuan Huang, Shun-Wei Liu, Wen-Yi Hung, Ken-Tsung Wong

**Affiliations:** 1 Organic Electronic Research Center, Ming Chi University of Technology, New Taipei City, 24031 Taiwan; 2 Department of Chemistry, National Taiwan University, Taipei, 10617 Taiwan; 3 Department of Optoelectronics and Materials Technology, National Taiwan Ocean University, Keelung, 20224 Taiwan; 4 Institute of Atomic and Molecular Science Academia Sinica, Taipei, 10617 Taiwan

**Keywords:** Organic LEDs, Structure elucidation

## Abstract

Two 2,7-dicyaonfluorene-based molecules 27-DCN and **27**-**tDCN** are utilized as acceptors (A) to combine with hexaphenylbenzene-centered donors (D) TATT and DDT-HPB for probing the exciplex formation. The photophysical characteristics reveal that the steric hindered **27-tDCN** not only can increase the distance of D and A, resulting in a hypsochromic emission, but also dilute the concentration of triplet excitons to suppress non-radiative process. The **27**-**tDCN**-based exciplex-forming blends exhibit better photoluminescence quantum yield (PLQY) as compared to those of 27-DCN-based pairs. In consequence, among these D:A blends, the device employing DDT-HPB:**27**-**tDCN** blend as the emissiom layer (EML) exhibits the best EQE of 3.0% with electroluminescence (EL) λ_max_ of 542 nm. To further utilize the exciton electrically generated in exciplex-forming system, two D–A–D-configurated fluorescence emitter DTPNT and DTPNBT are doped into the DDT-HPB:27-tDCN blend. The nice spectral overlap ensures fast and efficient Förster energy transfer (FRET) process between the exciplex-forming host and the fluorescent quests. The red device adopting DDT-HPB:**27**-**tDCN**:10 wt% DTPNT as the EML gives EL λ_max_ of 660 nm and maximum external quantum efficiency (EQE_max_) of 5.8%, while EL λ_max_ of 685 nm and EQE of 5.0% for the EML of DDT-HPB:**27**-**tDCN**:10 wt% DTPNBT. This work manifests a potential strategy to achieve high efficiency red and deep red OLED devices by incorporating the highly fluorescent emitters to extract the excitons generated by the exciplex-forming blend with bulky acceptor for suppressing non-radiative process.

## Introduction

Organic light-emitting diode (OLED) display technology with the merits of superior contrast ratios, true deep black and vivid colors, has become an integral part of our daily lives. In spite of the impressive efficiencies and lifetimes of the OLED devices fabricated with Ir-based emitters, efforts are underway to eliminate their dependence on rare metals to realize the goal of sustainable utilization of natural resources^1–3^. In recent, thermally activated delayed fluorescence (TADF) materials without the incorporation of metal atoms are emerging as next generation OLED emitters because of 100% external quantum efficiency (EQE) can be feasibly realized by the up-conversion of triplet excitons^4,5^. In order to have the smooth up-conversion process, a small singlet–triplet gap (∆*E*_ST_) is essential for a TADF chromophore that typically needs sophisticated synthesis, limiting the cost-effective production of emitting material for commercial display application. Alternatively, the exciplex-forming blend comprising physically blended electron-donor (D) and electron-acceptor (A) can also achieve full exciton utilization^6–9^. The exciplex formed by the intermolecular charge transfer at the D/A interface performs a small Δ*E*_ST_ due to the spatially well-separated highest occupied molecular orbital (HOMO) and lowest unoccupied molecular orbital (LUMO). The limited Δ*E*_ST_ ensures the effective reverse intersystem-crossing process (RISC) enabling the up-conversion of triplet exciton to singlet exciton. The optical energy gap of exciplex exciton is typically determined by the energy difference between the HOMO of D and the LUMO of A, rendering the emission energy tuning flexible. The D and A are typically also acting as hole- and electron-transporting material, respectively, leading to the reduced recombination barrier of exciton and thus the low turn-on voltage of the device^10–12^. In addition to serve as the emitting layer (EML), exciplex-forming system can also serve as the host enabling fast energy transfer to emitter with high photoluminescence quantum yield (PLQY), resulting in an extension of emission wavelength and improved device efficiency^13–18^. However, an important issue of the exciplex exciton is the low oscillator strength which results in the poor radiative rate and thus the relatively low PLQY due to the weak intermolecular electronic coupling. In addition, the dissociation of exciplex exciton is also the challenge that needs to be addressed^11^. Even though the complete D/A separation can result in obvious charge-transfer (CT) character leading to smaller ∆*E*_ST_ compared with the intramolecular TADF molecule. Another noteworthy attention is that the significant charge-transfer state of singlet and triplet proceeds a weaker spin-obit coupling (SOC) in El-Sayed rule, resulting in a slower RISC process. Several TADF researches have demonstrated that the original low-lying local-excited triplet state (^3^LE) which is enough close the charge-transfer state, can interact cooperatively with ^1^CT and ^3^CT state promoting the RISC process^19,20^. However, if the ^3^LE state is too low compared to the CT state, it can be counterproductive due to the participation of back electron-transfer process. Therefore, the sufficiently high triplet energy level in both D and A are indispensable.

In the pursuit of efficiency competition, a useful strategy is involved the insertion of the third layer into interfacial exciplex which decreases the probability of triplet-exciplex formation at interface for improving the exciplex PLQY. In 2016, Adachi et al*.* inserted the third layer of mCBP whit a thickness of 5 nm into the interfacial exciplex composed of m-TDATA as D and T2T as A, giving the blue-shifted emission due to the larger distance between D/A, and enhancing the device performance^21^. Su’s and coworkers also added 2 nm mCP as spacer layer into a bilayer exciplex system comprising TAPC as D and TmPyTz as A, giving the sky-blue emission and achieving better EQE_max_ up to 13%^22^. In the heterojunction of the exciplex, diluting the concentration of triplet exciton through well-mixing the third component with exciplex-forming components can suppress the deactivation of triplet–triplet annihilation (TTA) at the excited state, promoting the device efficiency and lifetime. Such as the silane-derivate, UGH3, doping into the TSBPA (D) :PO-T2T(A) blend can greatly improve the PLQY^23^. In 2022, Yan et al. reported that the addition of a spacer, mCP, into the exciplex-forming blend DMAC-DPS (D) :PO-T2T (A) gives the lower the concentration of triplet pairs to prevent TTA quenching, leading to reduce the non-radiative process^24^. Hence, a 53% increase of EQE for tri-component exciplex as compared to pure exciplex. In the presence of a third component, the concentration of the triplet exciton pair can be diluted, leading to the suppressed probability of bimolecular collision, and resulting in a weak non-radiative process, thus, representing a good strategy to perform the high efficiency and stability of exciplex-based device.

Another notable approach is the direct introduction of the bulky group into the molecular structure to prevent the π–π stacking of molecules that typically serves as an alternative transition pathway competing the radiative decay of exciplex. Larger steric effect of π-spacer not only reduces the concentration of triplet exciton, but further enhances the formation of CT state that can perform the better performance^23,25^. This strategy was verified by Hung’s study reporting the introduction of two bulky triphenylsilane groups onto the parent D, DTAF, to give DSDTAF. The green emission device employing the exciplex-forming blend DSDTAF (D):3N-T2T (A) achieved an improved EQE_max_ of 13.2% as compared to that (11.6%) of the parent DTAF:3N-T2T blend due to the steric effect of DSDTAF^25^. Similarly, Wang and coworkers reported a new acceptor material, TXO-P-Si, equipped with a bulky tetraphenylsilane pendant in a thioxanthen-9-one-10,10-dioxide (TXO) core. The intrinsic tetrahedral configuration of TOX-P-Si enables the formation of exciplex systems with high PLQYs when blended with carbazole-based donors.

For the quick conduct efficient electron-transfer from D to A, the deeper LUMO of the acceptor to afford enough driving force is necessary, together with the admirable electron-transporting ability to realize the carrier balance, leading to high device efficiency. In this regard, the introduction of cyano group onto the acceptor is a preferred strategy because of the electron-withdrawing ability and short conjugation of cyano group can lower the LUMO and avoid the overly extend π-conjugation for a low triplet state, respectively. For instance, the triazene-based acceptor, CN-T2T, with *m*-benzonitrile to end-cap the triazine core possesses a high triplet state (2.82 eV), serving as a useful A for many exciplex systems^10,13,14,17^. Along this line, Cao et al*.* reported a series of spiro[fluorene-9,9′-xanthene] (SFX)-based acceptors blending with donor TCTA to form exciplexes, in which the exciplex-forming blend with CN-substituted A gave a higher PLQY of 31% as compared to 15% for the SFX-based acceptor without CN-substitution^26^. More recently, Cao et al. adopted 9-phenylfluorene as a steric group of *N*-ethylcarbazole core appending a 2,7-dicyanofluorene via C9-linkage to develop a new acceptor^27^. The appearance of 9-phenylfluorene group significantly improve the efficiency of the device up to 25.1% EQE_max_ as compared to that (12.7%) of the device employing parent donor.

**Figure 1:**
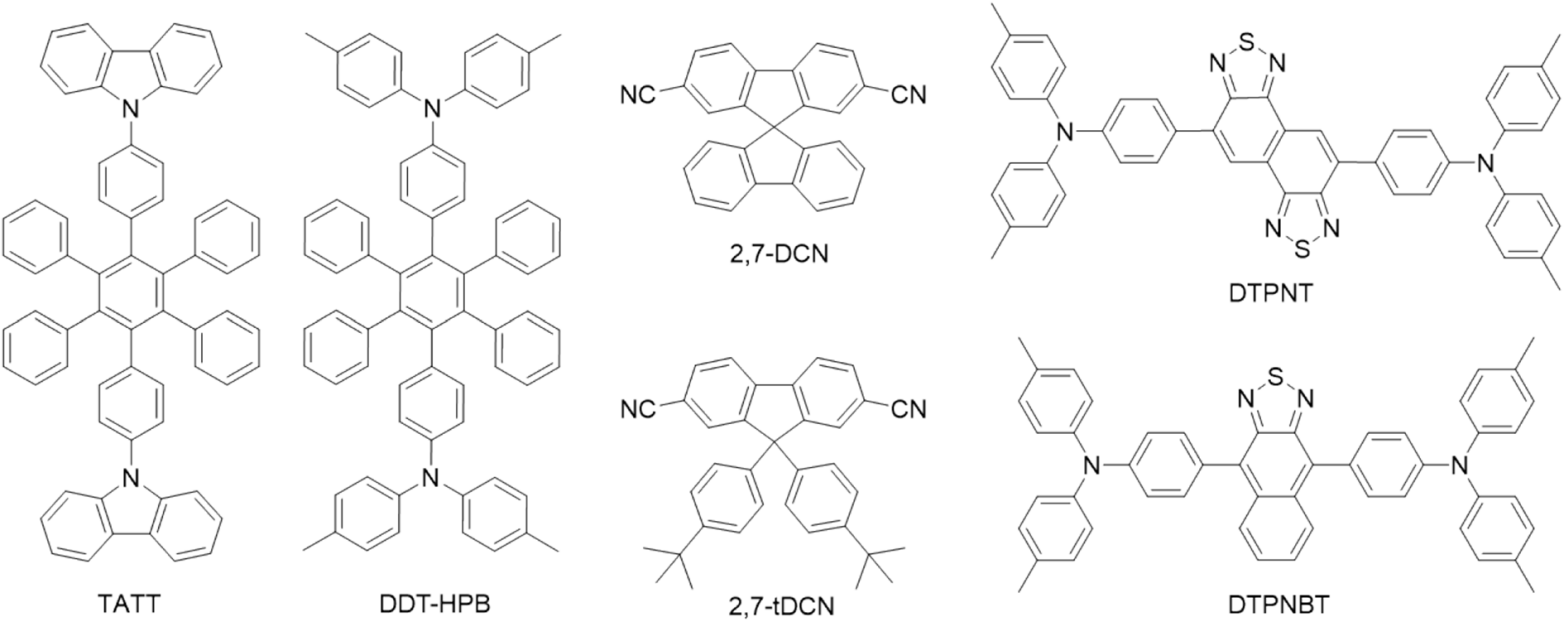
The molecular structure of TATT, DDT-HPB, 27-DCN, **27-tDCN**, DTPNT and DTPNBT.

In this work, two fluorene-based acceptors, 27-DCN and **27**-**tDCN** (Fig. 1), are reported to serve as the acceptors for exciplex-forming blends. The rigid spiro-configured 27-DCN and **27-tDCN** with two bulky *tert*-butyl phenyl groups are subject to blend with hexaphenylbenzene (HPB)-based bulky donors^14,28–30^. The exciplex of DDT-HPB:**27-tDCN** and TATT:**27-tDCN** blends gave the higher PLQY of 33% and 30% comparing to 19% and 18% for the DDT-HPB:27-DCN and TATT:27-DCN, implying the large steric effect of **27-tDCN** is beneficial for a superior exciplex-forming system. The device based on DDT-HPB:**27-tDCN** and TATT:**27-tDCN** as the exciplex EML gave EL emission of 542 nm and 514 nm and EQE_max_ of 3.0% and 2.0%, respectively. The further application of using the exciplex-forming DDT-HPB:**27-tDCN** blend as host of D-A-D-typed fluorescent emitters, DTPNT^18^ and DTPNBT^18^, was examined. The good spectral overlap between the emission of exciplex and the absorption of tow emitters results in an efficient Förster resonance energy transfer (FRET) for giving red emission. Notably, the complete energy transfer can be realized with 10 wt% doping concentration of DTPNT and DTPNBT, achieving the red OLED devices with the EL wavelength of 660 nm and 685 nm and EQE of 5.8% and 5.0%, respectively. These results reveal the potential strategy of using bulky fluorene-based acceptor for improving the propensity of exciplex formation in OLED device.

## Result and discussion

**Scheme 1:**
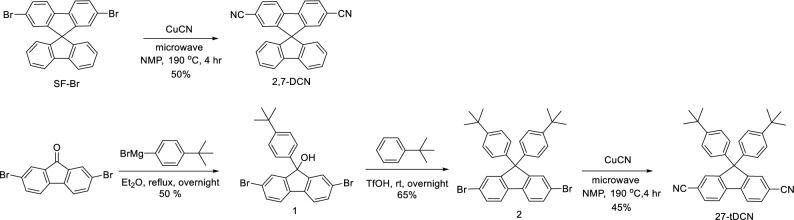
Synthesis route of 27-DCN and 27-tDCN.

The synthesis of 27-DCN and **27-tDCN** is illustrated in Scheme 1. The 27-DCN was synthesized through the Rosenmund-von Braun reaction of 2,7-dibromo-spirobifluorene^31,32^. For the synthesis of **27-tDCN**, the treatment of 2,7-dibromofluorenone with 4-tert-butylphenyl magnesium bromide yielded the intermediate alcohol 1^33,34^, which was subsequently underwent triflic acid-catalyzed Friedel–Crafts reaction with tert-butylbenzene to afford the 2,7-dibromofluorene intermediate 3^35,36^. Finally, the microwave assisted Rosenmund-von Braun reaction of 3 gave **27-tDCN** a yield of 45%. The thermal stability of the acceptors 27-DCN and **27-tDCN** was examined using thermogravimetric analysis (TGA) under nitrogen. The decomposition temperature (T_d_, 5% weight loss) of 27-DCN and **27-tDCN** are 306 °C and 330 °C (Table 1), indicating the rigidity of fluorene is beneficial for the sufficient thermal stability which is feasible for device fabrication by vacuum deposition.

**Table 1:** Photophysical, electrochemical, and thermal properties of molecules 27-DCN and **27-tDCN**.

Compound	λ^sol^_abs_^a^ (nm)	λ^film^_abs_^b^ (nm)	λ^sol^_PL_^a^ (nm)	λ^film^_PL_^b^ (nm)	E_g_^c^ (eV)	E_T_^d^ (eV)	HOMO^e^/LUMO^f^ (eV)	T_d_ (°C)
27-DCN	300,330	290,304	376	383,397	3.52	2.46	− 6.62/− 2.88	306
**27-tDCN**	300,331	301,330	361	359	3.62	2.49	− 6.52/− 2.78	330

^a^Measured in toluene solution (10^–5^ M).

^b^Measured in a neat film.

^c^Estimated from the onset of the UV–vis absorption curves in toluene.

^d^Estimated from the onset of the Phos spectra at 10 K in neat film.

^e^Calculated from the difference between the LUMO, and corresponding optical bandgap.

^f^HOMO level was calculated from oxidation potential as referred to the HOMO of ferrocene.

**Figure 2:**
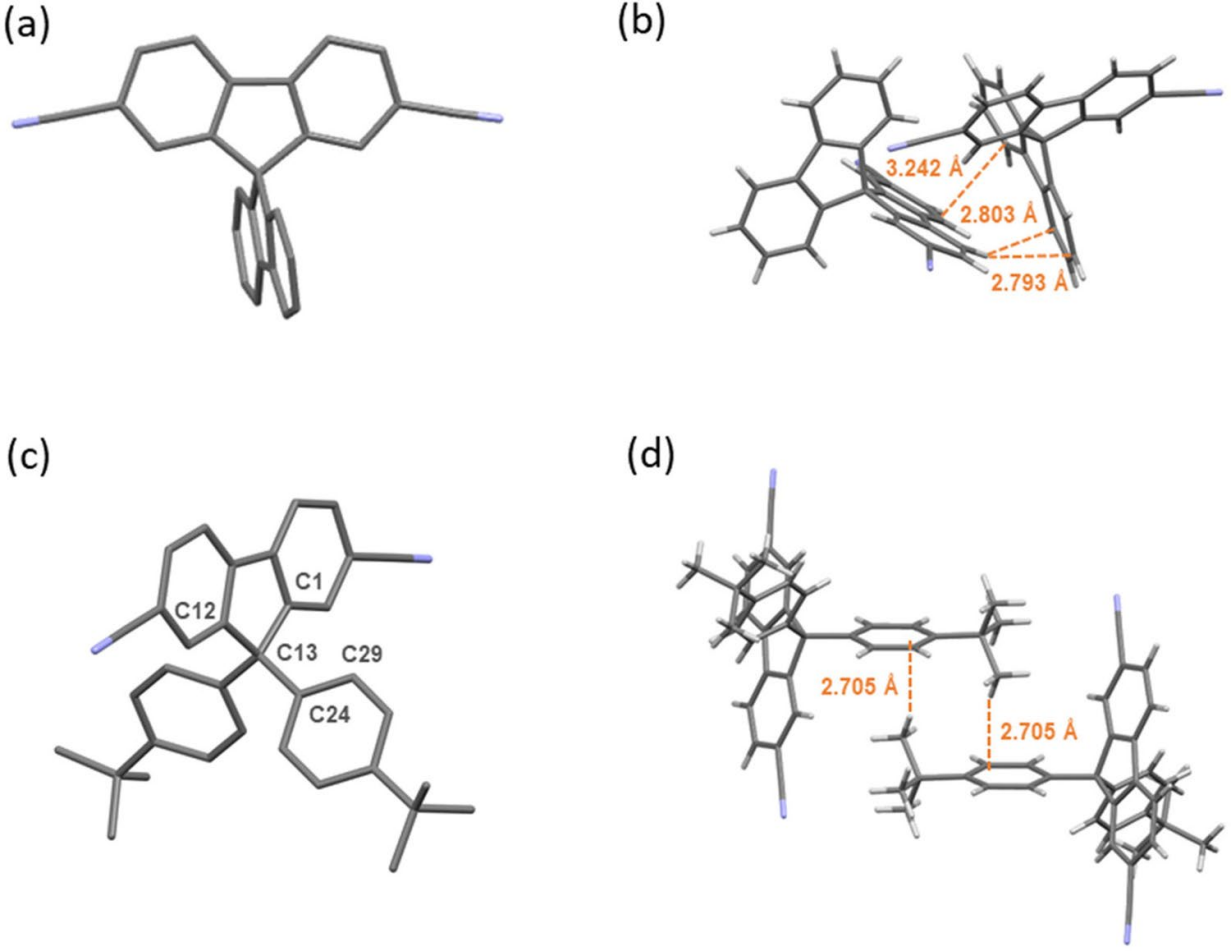
The X-ray structure and intermolecular interactions of 27-DCN and **27-tDCN** molecules.

The molecule structure and crystal packing of 27-DCN and **27-tDCN** are characterized by X-ray analysis of single-crystal (Fig. 2). Good quality crystals were obtained by slow diffusion of orthogonal solvents (dichloromethane/methanol). The 27-DCN and **27-tDCN** exhibit highly twisted structure due to the *sp*^*3*^-hybridized carbon bridge of fluorene^13,26^.The dihedral angle between two fluorene of 27-DCN is 80.7°, while the dihedral angle between fluorene and tert-butylbenzene (C12-C13-C24-C29) of **27-tDCN** is 89.3°, which is close to an orthogonal conformation. These conformations effectively block the p-conjugation extending from the CN-substituted fluorene to the peripheral fluorene and *t*-butylbenzene, giving both a relatively high triplet state. Interestingly, the dihedral angle (C1-C13-C24-C29) is 24.5°, allowing a weak electron coupling between fluorene and 4-butylbenzene of **27-tDCN**. The highly twisted conformation together with the different electronic features between fluorene and CN-substituted fluorene render the crystal packing of 27-DCN through several *π–π* interactions and C–H^**…**^ π interaction (Figs. 2c and S1a). Notably, the intermolecular interactions of **27-tDCN** are the C–H^**…**^*π* bonding between the *t*-butyl group and phenylene ring, leading to anti-parallel packing motif (Fig. 2d). In addition, two neighboring anti-parallel columns of **27-tDCN** crystals are linked by the weak non-conventional CN^**…**^H–Ph hydrogen-bonding (Fig. S1b). These subtle intermolecular interactions could be beneficial for facilitating carrier transport^37–39^.

**Figure 3:**
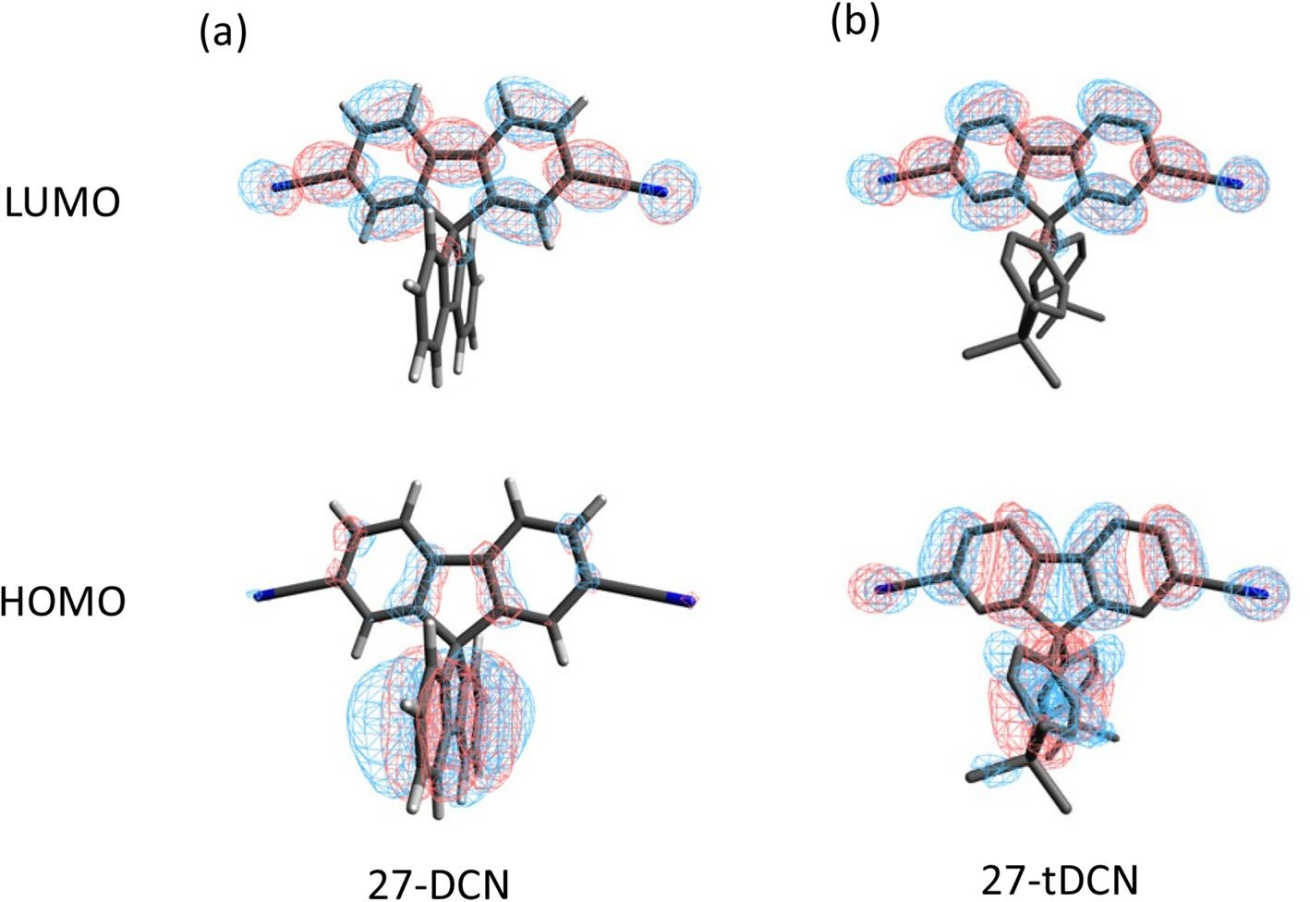
The HOMO and LUMO distributions of (**a**) 27-DCN and (**b**) **27-tDCN**.

To understand the correlation between molecular structure and physical properties, the DFT calculations of 27-DCN and **27-tDCN** were conducted. The ground state structure was optimized at a B3LYP/6-31G(d) level. As depicted in Fig. S2, the ground state structures of 27-DCN and **27-tDCN** exhibit the almost orthogonal configurations which are consistent with the observed X-ray structures. Compared to the HOMO of 27-DCN, which is mainly distributed on fluorene with limited contribution from dicyanofluorene branch (spiro-conjugation)^13^, the HOMO of **27-tDCN** is delocalized over the two tert-butylbenzene and dicyanofluorene, illustrating the through space conjugation from *tert*-butylbenzene to dicyanofluorene^40^ (Fig. 3), agreeing to the X-ray analyzed structures. This result reveals that the tert-butylbenzene peripherals of **27-tDCN** are not inert for electronically coupling with dicyanofluorene due to flexible conformation. Whereas the LUMOs of 27-DCN and **27-tDCN** are both distributed over the dicyanofluorene. The electrochemistry properties of 27-DCN and **27-tDCN** were studied by cyclic voltammetry (Fig. S3). The pertinent data are summarized in Table 1. 27-DCN and **27-tDCN** exhibit one quasi-reversible reduction process at − 1.95 V and − 2.02 V, respectively, which is attributed to the dicyanofluorene. Due to the stronger electron-donating ability of *tert*-butylbenzene, the reduction process of **27-tDCN** occurs slightly later as compared to that 27-DCN. By taking the ferrocene/ferrocenium (Fc/Fc^+^) redox couple as the standard, the corresponding LUMO energy level of 27-DCN and **27-tDCN** can be calculated as − 2.88 eV and − 2.78 eV, respectively. Then, the HOMO energy levels of 27-DCN and **27-tDCN** are deduced to be − 6.62 eV and − 6.52 eV by incorporating the optical energy gap.

**Figure 4:**
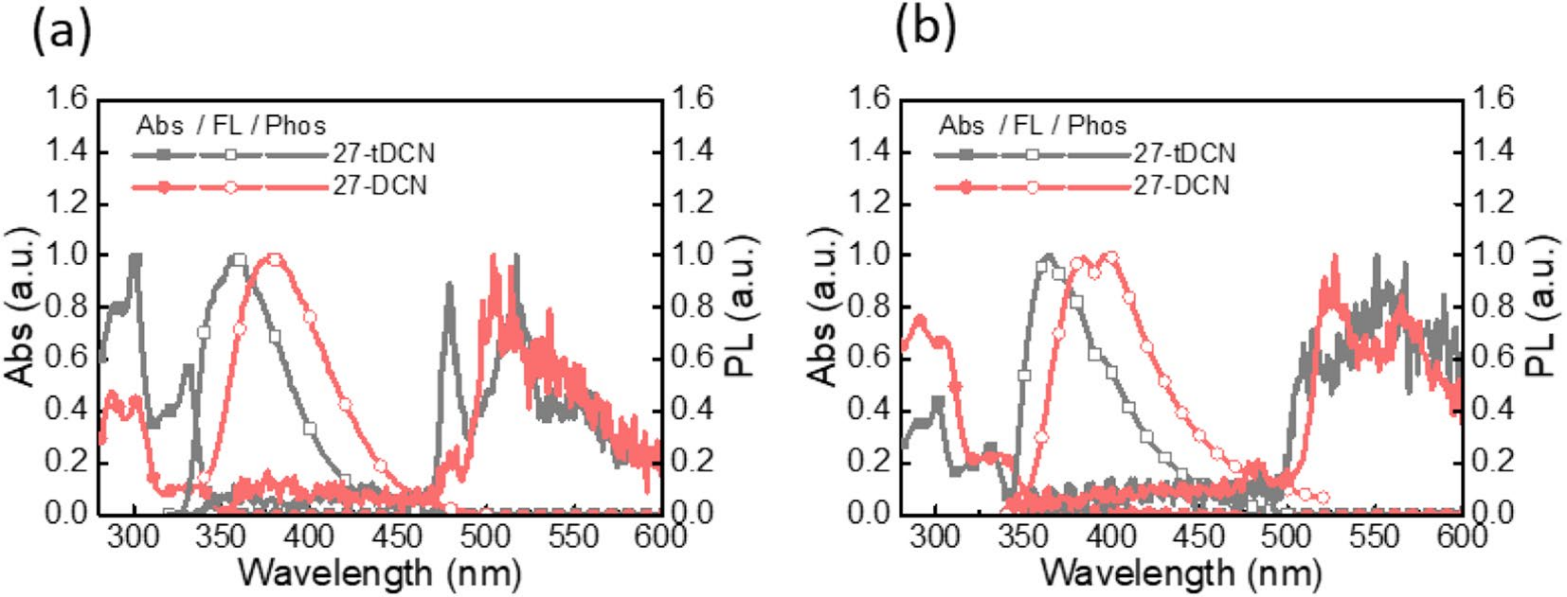
The absorption, photoluminescence, and phosphorescence spectra of 27-DCN and **27-tDCN** (**a**) in toluene solution (**b**) in neat film.

The ultraviolet–visible (UV–Vis) absorption, photoluminescence (PL) and phosphorescence spectra were conducted to realize the steady state photophysical properties of 27-DCN and **27-tDCN** in toluene solution and film. The corresponding photophysical data are summarized in Table 1. As depicted in Fig. 4a, both the 27-DCN and **27-tDCN** show an absorption peak centered at 300 nm and 330 nm, respectively, which is ascribed to *π*–π* transition. The emission peak centered at 376 nm for 27-DCN, and 361 nm for 27-tDCN was detected. The small Stokes shifts reveal the contribution from the rigid structure of fluorene skeleton. Surprisingly, 27-DCN shows a red-shifted emission as compared to that of **27-tDCN**, indicating a slightly extended *π*-conjugation due to the rigid spirobifluorene core of 27-DCN, whereas the flexible conformations of *t*-butylbenzene respect to dicyanofluorene diminish the contribution to π-conjugation. The triplet state of 27-DCN and **27-tDCN** was estimated as 2.54 eV and 2.65 eV by the onset of phosphorescence at 77 K in toluene solution. The absorption and emission profiles of 27-DCN and **27-tDCN** in neat film are similar to those observed in toluene solution (Fig. 4b). The singlet and triplet sate in neat film were determined based on the onset of the fluorescence (room temperature) and phosphorescence (10 K). The singlet state of 27-DCN and **27-tDCN** are 3.52 eV and 3.62 eV, while the triplet state of 27-DCN and **27-tDCN** are 2.46 eV and 2.49 eV, respectively.

**Figure 5:**
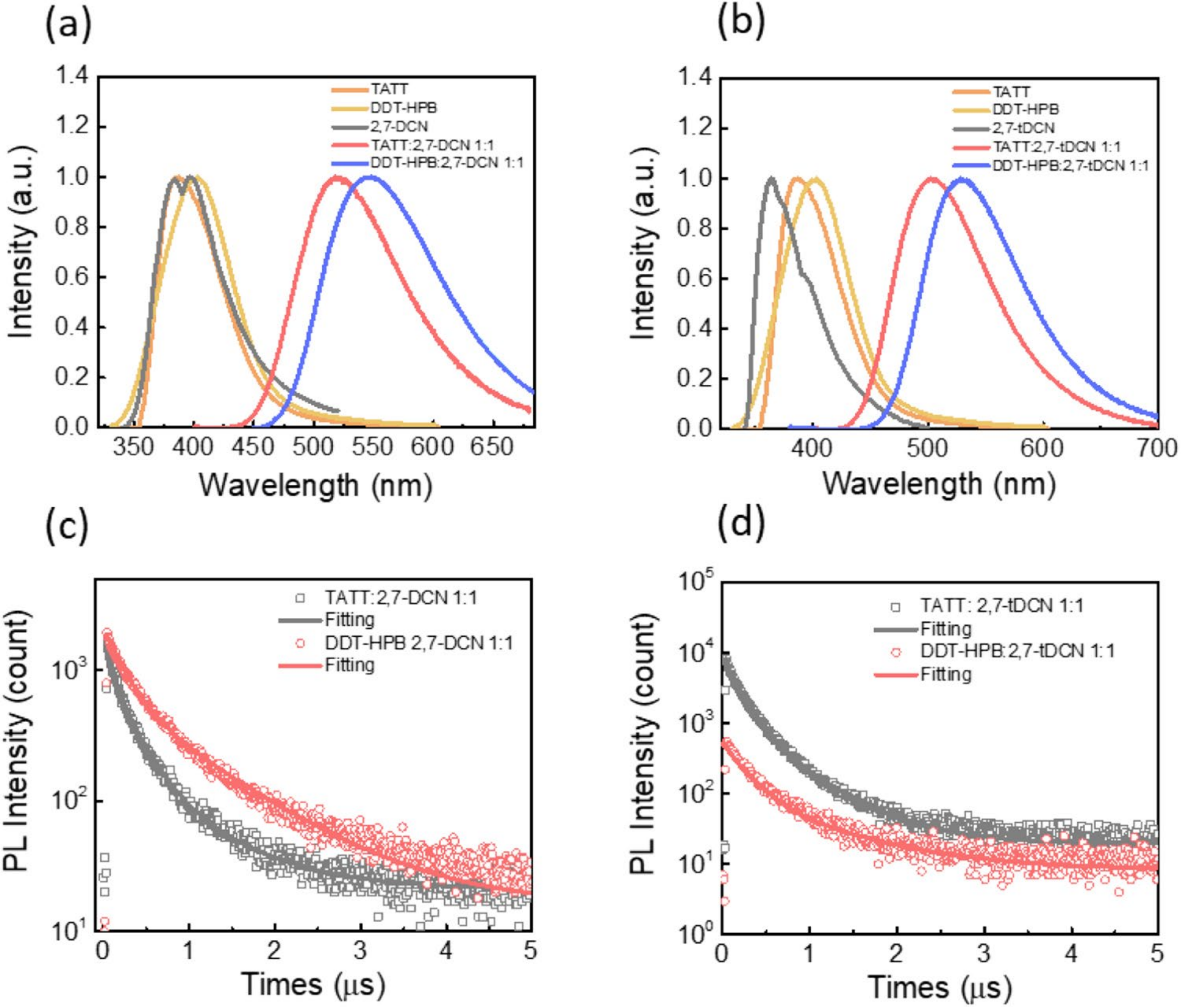
(**a**) Photoluminescence spectra of donor, acceptor 27-DCN neat film and D:A film. (**b**) photoluminescence spectra of donor, acceptor 27-tDCN neat film and D:A film, (**c**) the transient PL of TATT:27-DCN and DDT-HPB:27-DCN, (**d**) the transient PL of TATT:**27-tDCN** and DDT-HPB:**27-tDCN**.

For proceeding smooth charge transfer process, the appropriate HOMO/LUMO energy offset (0.4 eV) is required to afford enough driving force^7–10^. Therefore, the two bulky molecules, TATT and DDT-HPB, were selected as donors to probe exciplex formation with the acceptors 27-DCN and **27-tDCN**. The structure–property relationship of exciplex-forming blends with varying degrees of steric effect can be investigated. The energy alignments of the donors (TATT and DDT-HPB) and acceptors (27-DCN and **27-tDCN**) are depicted in Fig. S4. By vacuum deposition, four D:A blended films were prepared and examined. As depicted in Fig. S5, the absorption spectra of four blended films are integrated with individual donor and acceptor component without new absorption peak, indicating there is no electronic interaction between donor and acceptor. In contrast, the emissions of four blended films exhibit obvious bathochromic shifts without the residual emission from the donor and acceptor (Fig. 5a,b), revealing the signature of exciplex formation by intermolecular charge transfer process and inferring the electron transfer process are dominated in a low-lying excited state. As the ditolyamine of DDT-HPB is a stronger electron-donating group than diphenylamine of TATT, the stronger electronic coupling in the blend of DDT-HPB:27-DCN and DDT-HPB:**27-tDCN** leads to more stable charge-transfer state, resulting in the red-shifted emission centered at 548 nm and 531 nm, as compared to 519 nm and 502 nm observed for the blend of TATT:27-DCN and DDT-HPB:**27-tDCN**, respectively. It is worthy to note that the exciplex-forming blend comprising the acceptor **27-tDCN** with the larger steric hindrance to increase the distance between donor and acceptor gives a slightly blue-shifted emission which is consistent with the previous reports^21–24^.

The PLQY of TATT:27-DCN, TATT:**27-tDCN**, DDT-HPB:27-DCN and DDT-HPB:**27-tDCN** blends were measured using an integrating sphere to be 16%, 30%, 19% and 33%, respectively (Fig. S6). The superior PLQYs of TATT:27-tDCN and DDT-HPB:**27-tDCN** as compared to those blends with acceptor 27-DCN manifest the contribution of steric hindrance from the tert-butylbenzene groups of **27-tDCN** to increase the probability of exciton formation at the donor/acceptor interface^25^.

To further verify the kinetic decay process of theses exciplex excitons, the transient PL (TrPL) was conducted with an excitation wavelength of 300 nm. The TrPL spectra of four D:A blends as shown in Fig. 5 can be fitted with two exponential decayed model. The corresponding data are summarized in Table 2. The TrPL signals of TATT:27-DCN and DDT-HPB:27-DCN blends were fitted with the fast lifetime of 288.0 ns and 210.2 ns, respectively, which is attributed to the prompt fluorescence, and the slower lifetime of 3.4 μs and 1.2 μs, which is considered to be the delayed fluorescence. Besides, two-exponential lifetime of 149.3 ns and 0.5 μs was fitted to the TrPL detected for the TATT:**27-tDCN** blend, while the blended film of DDT-HPB:**27-tDCN** shows the obvious prompt and delay lifetime of 238.0 ns and 1.0 μs, respectively.

**Table 2:** The TrPL characteristics of the donor:acceptor blended films.

Donor:acceptor	λ_max_ (nm)	Lifetime				Δ*E*_ST_ (meV)
		A_1_	τ_1_ (ns)	A_2_	τ_2_ (μs)	
TATT:27-DCN	519	0.82	288.0	0.18	3.4	11
DDT-HPB:27-DCN	548	0.83	210.0	0.16	1.2	13
TATT:**27-tDCN**	502	0.81	149.3	0.18	0.5	11
DDT-HPB:**27-tDCN**	531	0.83	238.0	0.16	1.0	13

According to the previous TADF kinetic studies^10,14^, assuming the pre-equilibrium between S_1_ and T_1_ states, the time-resolved fluorescence intensity, [S_1_]_f_, can be expressed using the following equation:

$$[{\text{S}}1]{\text{f}}=\mathrm{ I}0 \left\{\frac{{k}_{risc}}{{k}_{isc}+{k}_{isc}}\right.{e}^{\frac{-t}{{\tau }_{1}}}+\left.\frac{{k}_{isc}}{{k}_{isc}+{k}_{risc}}{e}^{\frac{-t}{{\tau }_{2}}}\right\}$$

where I_0_ is a proportional constant merging both radiative decay rate constant of the exciplex emission and instrument factor, τ_1_ and τ_2_ are the lifetime of the fast and slow decay component, respectively. Following this equation, the equilibrium constant K_eq_ = K_isc_/K_risc_ can be obtained by the ratio of the pre-exponential factor. Then, the K_eq_ of the TATT:27-DCN, DDT-HPB:27-DCN, TATT:**27-tDCN** and DDT-HPB:**27-tDCN**. blend can be deduced to be 5.1, 4.5, 4.5 and 5.1, respectively. Combining the Δ*E*_ST_-K_eq_ relationship formula, Δ*E*_ST_ = − RTln (K_eq_/3), where the factor of 3 stands for the triplet degenerate states, the Δ*E*_ST_ data of the blend film is calculated to be 11 meV, 13 meV, 11 meV and 13 meV for TATT:27-DCN, DDT-HPB:27-DCN, TATT:**27-tDCN** and DDT-HPB:**27-tDCN**, respectively. Since the K_RISC_ rate is proportional to the reciprocal of ∆*E*_ST_, the small ∆*E*_ST_ demonstrates that the exciplex excitons can undergo RISC process at room temperature, giving the delayed fluorescence. However, the PLQYs of exciplex-forming blends with 27-DCN are slightly lower as compared to those blends with 27-tDCN, inferring the distance between donor and acceptor of the 27-DCN-based blend is shorter, resulting in TTA process with high concentration of triplet exciton pair and charge dissociation at D/A interface competing the radiative transition. Along this line, the larger steric hindrance of **27-tDCN** can perform the weaker exciton dissociation and dilute the concentration of exciton pair, preventing the other non-radiative pathways for giving the better PLQY of DDT-HPB:**27-tDCN**.

**Figure 6:**
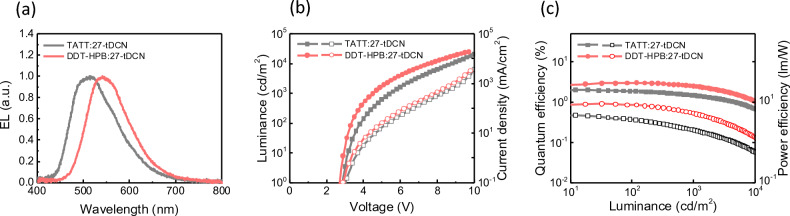
Characterization of exciplex-based OLED devices. (**a**) Electroluminescence of exciplex device. (**b**) Current density–voltage–luminance (J–V–L) characteristics. (**c**) External quantum efficiencies (EQE) and power efficiencies (PE) as a function of luminance.

According to the PL spectra of the blend films, two blend films DDT-HPB:**27-tDCN** and TATT:**27-tDCN** were examined as the EML of the OLED device configured as ITO/4 wt% ReO_3_:donor (60 nm)/donor (10 nm)/donor:**27-tDCN** (30 nm)/**27-tDCN**(10 nm)/CN-T2T(40 nm)/Liq/Al were fabricated. The 4 wt% ReO_3_:donor was used as a hole injection layer (HIL), while 8-hydroxyquinolinolato-lithium (Liq) modified Al was selected as the electron injection layer (EIL). The donor and CN-T2T were chosen as the hole- and electron- transporting layer, respectively. **27-tDCN** was employed as both the acceptor and hole-blocking layer. Figure 6 shows the EL characteristics of the devices. As shown in Fig. 6a, the EL λ_max_ peaks at 542 nm and 514 nm for the device employing DDT-HPB:**27-tDCN** and TATT:**27-tDCN** blend as the EML, respectively, which are slightly bathochromic shift as compared with the PL spectra. From the current density–voltage–luminance (J–V–L) curves (Fig. 6b), two devices apparently exhibit low turn-on voltage, indicating the smooth carrier injection barrier due to the appropriate energy level alignments (Fig. S4). For the device based on DDT-HPB: **27-tDCN** give the peak EQE, current efficiency (CE), and power efficiency (PE) of 3.0%, 9.2 cd A^−1^ and 9.1 l m W^−1^, compared to 2.0%, 4.8 cd A^−1^ and 4.7 l m W^−1^ for the device based on TATT:**27-tDCN**. The obtained inferior EQE_max_s are in line with the relatively low PLQYs of the EMLs (Table 3). Since the energy offset between local-excited triplet state (^3^LE) of 27-tDCN as closed as the ^1^CT of exciplex with value of 0.33 eV for TATT:**27-tDCN** and 0.20 eV for DDT-HPB: **27-tDCN**, the spin-flip process at excited state can arise resulting in a deactivation transition between ^1^CT and ^3^LE aaccording to El-Sayed rule. The process is also well-known as back electron transfer. In particular, the energy offset less or equal to 0.2 eV is beneficial to promote the spin-flip process that excitons have the better probability of up-conversion from ^3^LE to ^1^CT preventing the exciton was trapped in low-lying triplet state and maintaining the efficiency of yielding photon. This result reveals that EML of DDT-HPB:**27-tDCN** presents better device performance than TATT-based exciplex.

**Table 3:** Electroluminescence data of OLEDs based on exciplex host and dopants.

EML	EL_max_ (nm)	V_on_^a^ (V)	EQE_max_^a^ (%)	CE_max_ (cd A^−1^)	PE_max_ (lm W^−1^)	at 10^3^ nits (%)	CIE_max_ (x, y)
TATT:27-tDCN 1:1	514	2.7	2.0	4.84	4.72	1.46	(0.29, 0.47)
DDT-HPB:27-tDCN 1:1	542	2.6	3.0	9.22	9.09	2.51	(0.38, 0.55)
DDT-HPB:27-tDCN:DTPNT 1:1:10 wt%	660	2.8	5.8	2.32	2.61	1.11	(0.67, 0.32)
DDT-HPB:27-tDCN:DTPNBT 1:1:10 wt%	685	2.6	5.0	0.74	0.71	1.30	(0.69, 0.30)

**Figure 7:**
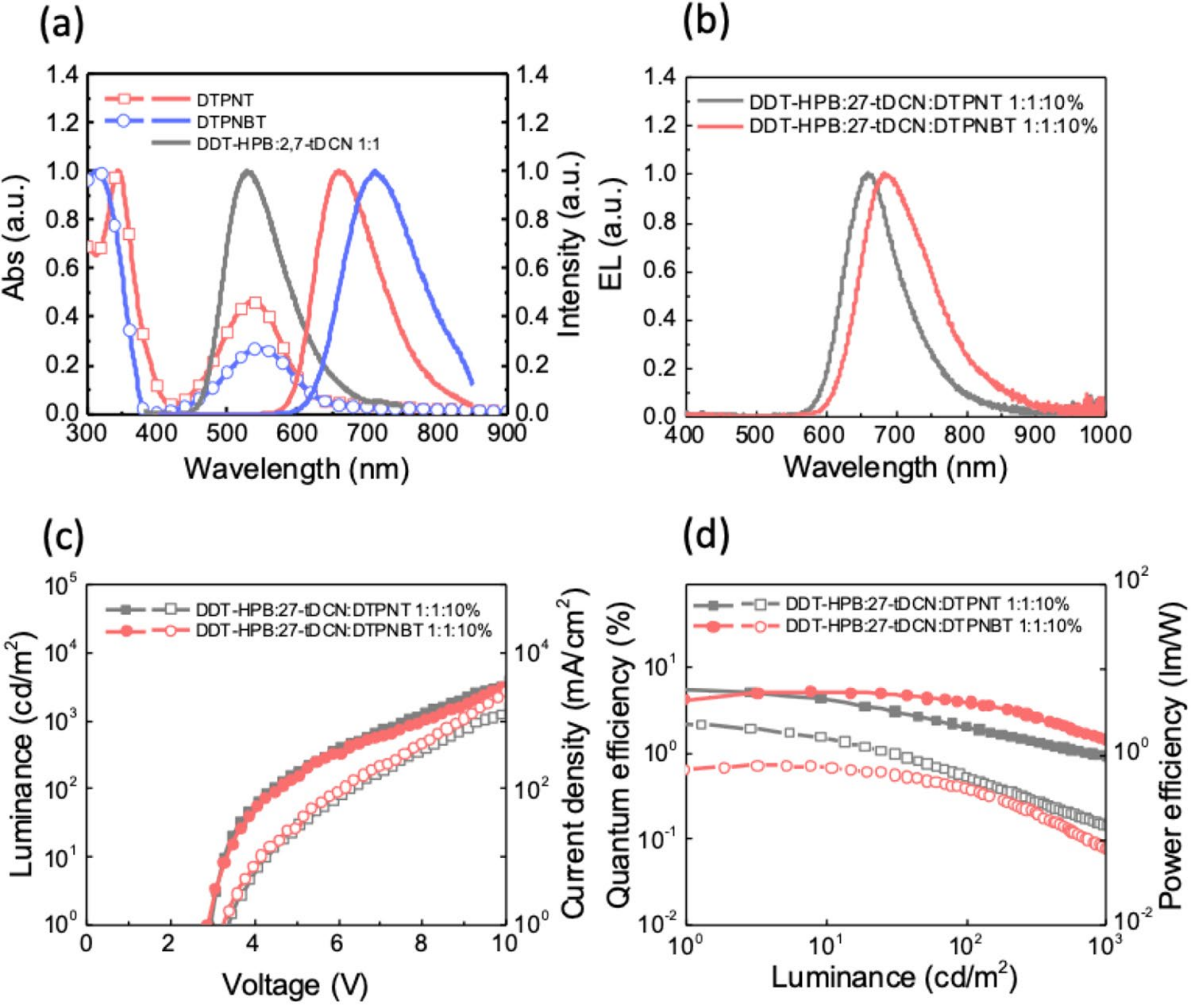
(**a**) The overlap of UV–Vis absorption and Photoluminescence spectra. (**b**) Electroluminescence spectra (**c**) Current density–voltage–luminance (J–V–L) characteristics. (**d**) External quantum efficiencies (EQE) and power efficiencies (PE) of the 10% dopant DTPNT and DTPNBT in DDT-HPB:**27-tDCN**.

To extract the exciplex excitons formed at the D/A interface, two D-A-D-type fluorescence emitters DTPNT and DTPNBT were introduced into the exciplex-forming DDT-HPB:**27-tDCN** host as the emissive dopant. The nice spectral overlaps (Fig. 7a) ensure that the exciplex exciton can efficiently transfer to the emitters through fast FRET, leading to the enhanced device EQEs. The films of the DDT-HPB:**27-tDCN** blend as host doped with 10 wt% DTPNT and DTPNBT exhibit significantly red-shifted emission centered at 664 nm and 699 nm (Fig. S7a), respectively. The PLQY of DDT-HPB:**27-tDCN** blend doped with 10 wt% DTPNT and DTPNBT is 86% and 58%, respectively (Fig. S6). Clearly, the PLQYs are largely improved, implying the FRET process dominates the kinetic relaxation process at low-lying charge-transfer state of the exciplex-forming host. To understand the corresponding relaxation processes of the doped films at excited state, the TrPL of 10 wt% emitter doped exciplex system was probed as shown in Fig. S7b. The resolved lifetimes of DDT-HPB:**27-tDCN**:10 wt% DTPNT are 8.3 ns and 154 ns, whereas the fitted lifetimes of DDT-HPB:**27-tDCN**:10 wt% DTPNBT are 9.8 ns and 306 ns. Both prompt and delayed lifetimes are significantly decreased as compared to those of the exciplex-forming host, indicating that the major kinetic relaxation pathway of the exciplex at the low-lying excited state is the FRET process. To explore the gain efficiency of fluorescent emitter-doped device, the EL characteristics of the device with different doped ratio were examined. For suppressing the Dexter energy transfer process to the triplet state of fluorescent dopant, 1 wt% DTPNT and DTPNBT were introduced as dopant of the exciplex-hosted device, giving the EOE_max_ of 5.0% and 3.0%, respectively, with the severe residual emission of exciplex host, indicating the incompletely energy transfer between the exciplex-forming host and the fluorescent dopants (Figs. S8 and S9). Upon increasing the dopant concentration to 5%, sufficient energy transfer from exciplex to emitter, can be achieved. Surprisingly, the maximum efficiency of the fluorescent emitter doped-device was achieved when the dopant concentration reaches to 10 wt%. The EQE_max_ of DTPNT- and DTPNBT-doped device is 5.5% and 5.0% with the EL wavelength centered at 660 nm and 685 nm, respectively **(**Fig. 7). The efficiency improves upon the increase of the dopant concentration from 5 wt% to 10 wt%, implying larger steric hindrance of exciplex can avoid contact with each D-A-D-type emitter in space thereby suppressing the intermolecular interactions, resulting in a low non-radiative rate which is beneficial for harvesting more photon. (Table 3) The result reveals the potential of introducing bulky steric hindrance for exciplex-forming systems, which can accommodate a higher concentration of dopant to extend the emission wavelength into near-infrared region.

In order to achieve practical applications of OLEDs, addressing device stability during continuous operation is a critical concern. Consequently, the operational lifetimes of both exciplex-based devices and OLED devices were evaluated under a constant current density of J = 10 mA/cm^2^. Notably, the stability of the device with 10 wt% DTPNBT doped into exciplex co-host system exhibit the better lifetime (ca. 890 h) compared with 69 h of 10 wt% DTPNT employing the exciplex co-host device. It is crucial to highlight that the DTPNBT device demonstrates a significantly extended operational lifetime, underscoring the promising potential of exciplex-hosted deep-red OLEDs for future practical commercialization.

## Conclusion

In summary, two dicyanofluorene-based acceptors 27-DCN and **27-tDCN**, in which tert-butylbenzene was introduced into **27-tDCN** to create steric hindrance, were examined as acceptors for new exciplex-forming systems. The electrochemical and photophysical properties of both acceptors were characterized. The highly twisted structure observed from the crystal structures and DFT calculations result in the high triplet state of 2.69 eV for 27-tDCN and 2.68 eV for 27-DCN. Two acceptors blend with HPB-based donors, DDT-HPB and TATT, which also have a highly twisted configuration of structure, give red-shifted emissions and characteristics of delay fluorescence, indicating the successful formation of exciplex. The 27**-tDCN**-based exciplex-forming blends exhibit blue-shifted emission and better PLQY because the large steric interactions at the D/A interface reduces the concentration of triplet exciton pair which can suppress the annihilation behavior. However, the further applications of DDT-HPB:**27-tDCN** and TATT:**27-tDCN** blends as the EML afford the device with EQE_max_ of 3% and 2%, respectively. To extract more the exciplex excitons in the device, two D-A-D-typed fluorescence emitters DTPNT and DTPNBT were doped. The absorption spectra of DTPNT and DTPNBT present greatly overlap with the EL spectra of the DDT-HPB:**27-tDCN**, ensuring efficient FRET process. Therefore, the impressively high PLQY of 86% and 58% were observed when the 10 wt% DTPNT and DTPNBT were respectively doped into DDT-HPB:**27-tDCN** co-host. Lastly, the red OLED device employing the 10 wt% DTPNT and DTPNBT doped in the DDT-HPB:**27-tDCN** exciplex-forming host achieves EQE_max_ of 5.8% with EL λ_max_ centered at 660 nm and 5.0% with EL λ_max_ of 685 nm, respectively. This work manifests a practical strategy to fabricate high efficiency red and deep red OLED device can be realized through efficient FRET between the high fluorescence-based emitters and the exciplex-forming host systems with bulky acceptor for suppressing non-radiative process.

### Supplementary Information

Supplementary Information.

## Data Availability

The datasets used and/or analysed during the current study available from the corresponding author on reasonable request.
