# Supplementary material for: Exciplex-forming cohost systems with 2,7-dicyanofluorene acceptors for high efficiency red and deep-red OLEDs
Source: Sci Rep. 2024 Jan 30;14:2458. doi: 10.1038/s41598-024-52680-6 (PMC10827723; doi:10.1038/s41598-024-52680-6)

Supporting Information

Exciplex-forming Cohost Systems with amorphous Fluorene-based Acceptor for Highly Efficient Red and deep-Red OLEDs

Yi-Sheng Chen^1,2^, I-Hung Lin^3^, Hsin-Yuan Huang ^3^, Shun-Wei Liu^1^, Wen-Yi Hung^3,^*, Ken-Tsung Won^2,4^*,

^1^ Organic Electronic Research Center, Ming Chi University of Technology, New Taipei City 24031, Taiwan.

^2^ Department of Chemistry, National Taiwan University, Taipei 10617, Taiwan

^3^ Department of Optoelectronics and Materials Technology, National Taiwan Ocean University, Keelung 202, Taiwan

^4^Institute of Atomic and Molecular Science Academia Sinica, Taipei 10617, Taiwan.

E-mail: [kenwong@ntu.edu.tw](mailto:kenwong@ntu.edu.tw) (Ken-Tsung Wong); [wenhung@mail.ntou.edu.tw](mailto:wenhung@mail.ntou.edu.tw) (Wen-Yi Hung)

**General Method**

**Material Characterization**

All the chemicals and reagent were employed from commercial sources. Solvents for chemical analysis and reaction were purified by distillation before use. NMR using deuterium substituted solvent as an internal reference.

**Physical properties measurement**

Optical absorption experiments were conducted in solution by JASCO V-670 spectrophotometer. Photoluminescence spectra were measure using a spectrophotometer (HITACHI F-4500 fluorometer) equipped with a liquid nitrogen attachment at room temperature and 77K

**Electrochemical properties measurement**

Electrochemical measurements were investigated by an electrochemical analyser (CHI1619B, CH Instruments) employing Ag/Ag^+^ (Ag/0.01M AgNO_3_) as the reference electrode, a Pt wire as the counter-electrode, a glassy carbon electrode as the working electrode and an internal ferrocene/ferrocenium (Fc/Fc^+^) redox couple. Molecules for reduction potential were carried out in THF (0.01 M) containing 0.1 M tetrabutylalummonium hexafluorophosphate (nBuNPF_6_) as a supporting electrolyte with argon before conducting the experiments.

**Thermal properties measurement**

Thermogravimetric analysis (TGA) was measurement under a nitrogen atmosphere at a heating rate of 10 °C/min on a platinum pan via a TA Instruments Q500 TGA (V20.13 Build 39)

**Method for Theoretical Calculation**

Density function theory (DFT) calculations were conducted in gaseous state B3LYP/6-31+G(d) level by using a Gaussian 16 program package through Taiwania 1, build by the National Applied Research Laboratories, Taiwan.

**PLQY and TrPL lifetime measurements**

PLQYs of thin films were detected using quantum yield spectrometer (Hamamatsu C9920-02). During the PLQY measurements, the integrating sphere was purged with prue and dry nitrogen to keep the environment inert. The time-resolved studies were performed using a time-correlated single photon counting (TCSPC) system (TimeHarp 260, PicoQuant) with the pulse LED at 285 nm (PLS289, PicoQuant) as the photoexcitation light source.

**OLED fabrication**

All chemicals were purified through vacuum sublimation prior to use. The OLED were fabricated through vacuum deposition of the materials at 10^-6^ torr onto the ITO-coated glass substrates having a sheet resistance of 15 Ω sq–1. Prior to use the ITO surface was cleaned ultrasonically, *i.e.* With acetone, methanol, and deionized water in sequence and finally with N_2_ plasma. The deposition rate of each organic material was 1-2 Å∙s^–1^. The J-V-L (R) characteristics of the devices were measured simultaneously in a glovebox. A programmable source measurement unit (2614B, Keithley) was used as a driving source of the device while the light intensity was measured by a calibrated silicon detector. EL spectra were measured using a photodiode array (Ocean Optics USB2000+).

Synthesis of 1

To a two-neck round-bottom flask was added 2,7-dibromo-9*H*-fluoren-9-one (6.7g, 20.0 mmol) and the flask was evacuated and purged with argon gas, then ether (200.0 mL) was added. The 4-tert-butylphenyllmagnesium bromide (1 M in ether, 24.0 mL,24.0 mmol) was added at 0 ^o^C. The mixture was refluxed overnight. After cooling temperature, the mixture was extracted with ether and washed with brine. The organic solution was dried over MgSO_4_ and concentrated to give yellow residue. The crude product was purified by the column chromatography on silica gel (hexane/DCM = 2/3) to afford the title compound as a beige solid (4.70 g, 50%).^1^H NMR (400 MHz, *d*_6_-DMSO) δ 7.82 (d, J = 8 Hz, 2H), 7.59 (d, J = 8 Hz, 2H) 7.37 (s, 2H), 7.30 (d, J = 8 Hz, 2H), 7.16 (d, J = 8 Hz, 2H), 6.58 (s, 1H) 1.23 (s, 9H) ^13^C NMR (101 MHz, *d*_6_-DMSO) δ 153.3, 149.6, 140.5, 137.4, 127.5, 125.1, 124.7, 122.6, 121.3, 82.3, 34.1, 31.1MS(m/z, MALDI-TOF/TOF) Calcd for C_23_H_20_Br_2_O 471.9860, found 472.0508.

Synthesis of DDT-HPB

Synthesis of 2

The mixture 1 (2.0 g, 4.2 mmol) and tert-butylbenzene (50.0 mL, 323.0 mmol) was treated with TfOH (0.75 mL) at 0 ^o^C overnight. Then, the mixture was extracted with ether and washed with brine and saturated NaHCO_3_ aqueous. The organic solution was dried over MgSO_4_ and concentrated to give the crude product as brown solid. The cured product was purified by the column chromatography on silica gel with eluent of hexanes to afford the title compound as a white solid (1.60 g, 65%). ^1^H NMR (400 MHz, *d*_6_-DMSO) δ 7.92 (d, J = 8 Hz, 2H), 7.61 (d, J = 4 Hz, 2H) 7.57 (s, 2H), 7.32 (d, J = 8, 4H), 7.01 (d, J = 8 Hz, 4H), 1.24 (s, 18H) ^13^C NMR (101 MHz, CDCl_3_) δ 153.6, 150.0, 142.4, 130.9, 129.7, 127.8, 125.1, 121.6, 65.7, 34.6, 31.5

Synthesis of 27-tDCN

The mixture of compound 2 (2.30 g, 4.00 mmol), and copper(I) cyanide (3.20 g, 36.00 mmol) was dissolved in N-Methyl-2-pyrrolidone (40 mL). The reaction mixture was treated with microwave at 190 ^o^C for 4 h. After cooling temperature, the mixture was extracted with ether and washed with brine. The organic solution was dried over MgSO4 and concentrated to give the crude product as brown liquid. The crude product was purified by the column chromatography on silica gel (hexane/DCM = 1/1) and reprecipitation from DCM/MeOH to afford the title compound as a white solid (0.87 g, 45%). ^1^H NMR (400 MHz, CD_2_Cl_2_) δ 7.86 (d, J = 8 Hz, 2H), 7.70 (s, 2H) 7.67 (dd, J = 8, 1.6 Hz, 2H), 7.25 (dd, J = 8.4, 2 Hz, 4H), 7.00 (d, J = 8.4 Hz 4H), 1.27 (s, 18H) ^13^C NMR (101 MHz, CD2Cl2) δ 153.1, 150.6, 142.4, 139.9, 131.9, 130.3, 127.3, 125.7, 121.7, 118.9, 112.4, 65.1, 34.5, 31.2. MS (m/z, ESI-Q-TOF) Calcd for C_35_H_33_N_2_ 481.2638, found 481.2619.

**Figure S1** the crystal packing of (a) 27-DCN and (b) 27-tDCN

**Figure S2** the optimized structure of (a) 27-DCN and (b) 27-tDCN

**Figure S3** The cyclic voltammogram of (a) 27-DCN and (b) **27-tDCN** in TFH containing 0.1 M TBAPF_6_.


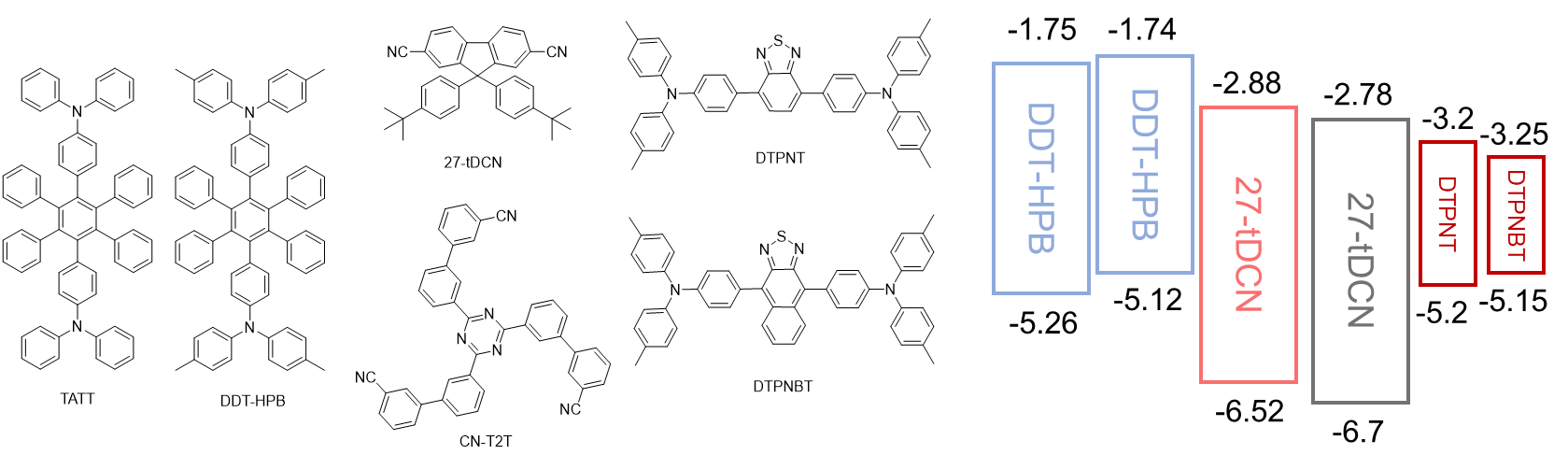


**Figure S4** energy alignment of materials

**Figure S5** the absorption spectra


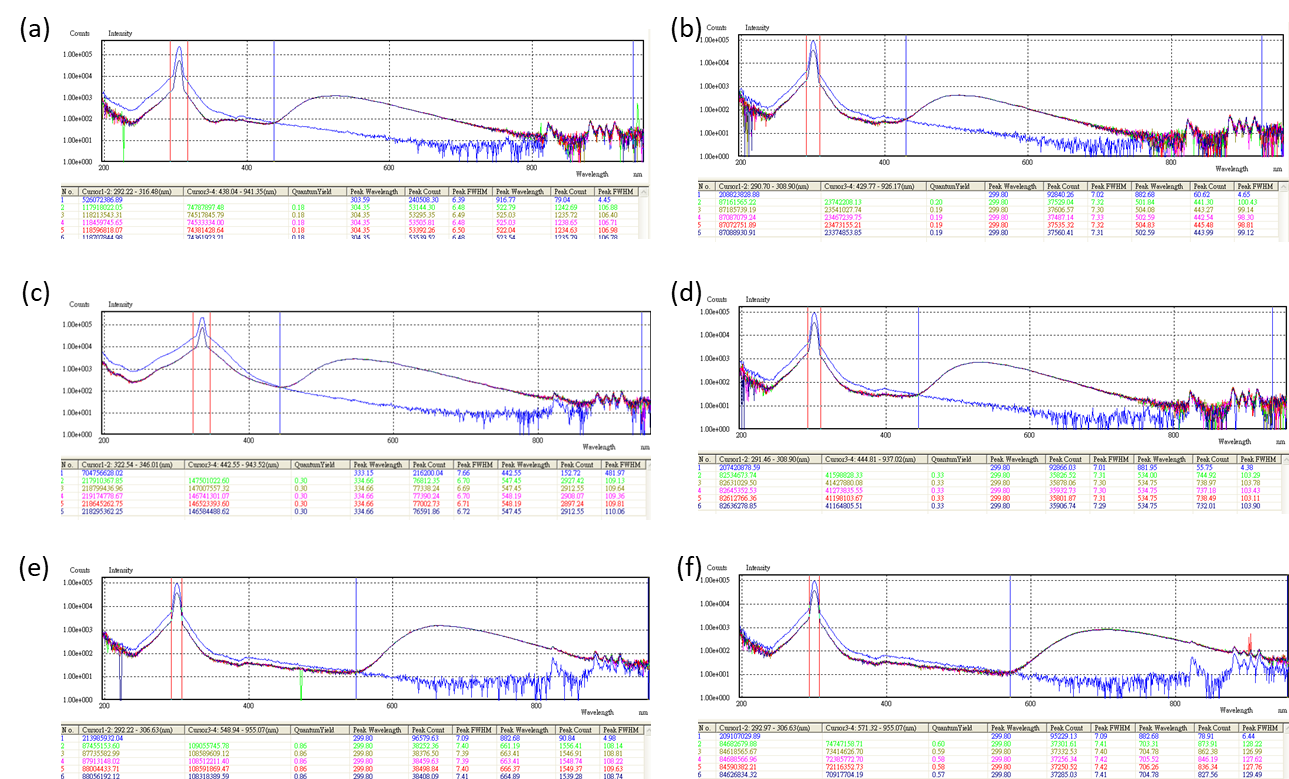


**Figure S6** PLQY of (a) TATT:27-DCN (b) DDT-HPB: 27-DCN (c) TATT:27-tDCN (d) DDT-HPB:27-tDCN (e) TATT:27-tDCN 10 % DTPNT (f) DDT-HPB: 27-tDCN:10% DTPNBT


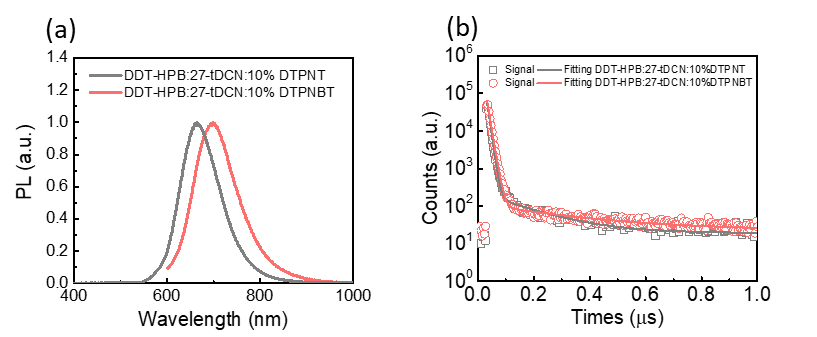


**Figure S7** (a) The PL spectra and (b) TrPL spectra of DDT-HPB:27-tDCN:10% DTPNT (black) and DDT-HPB:27-tDCN:10% DTPNBT (red)


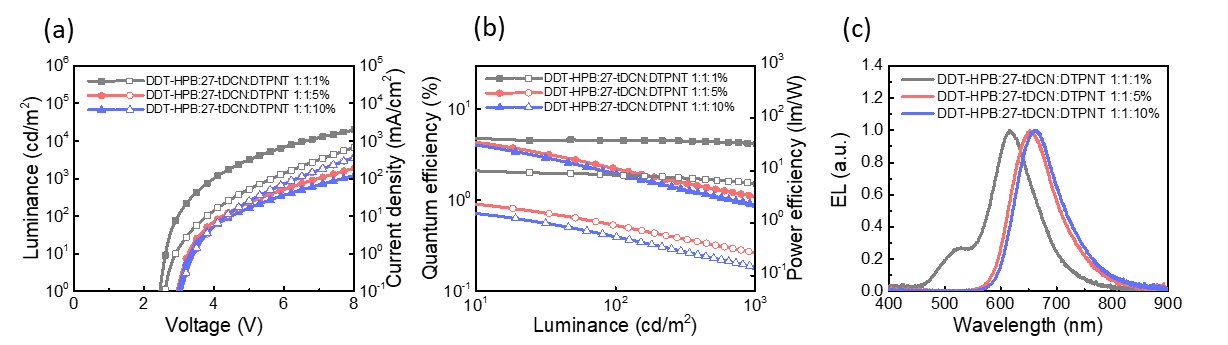


**Figure S8** different concentration of dopant DTPNT into blend film in OLED electronic characteristic


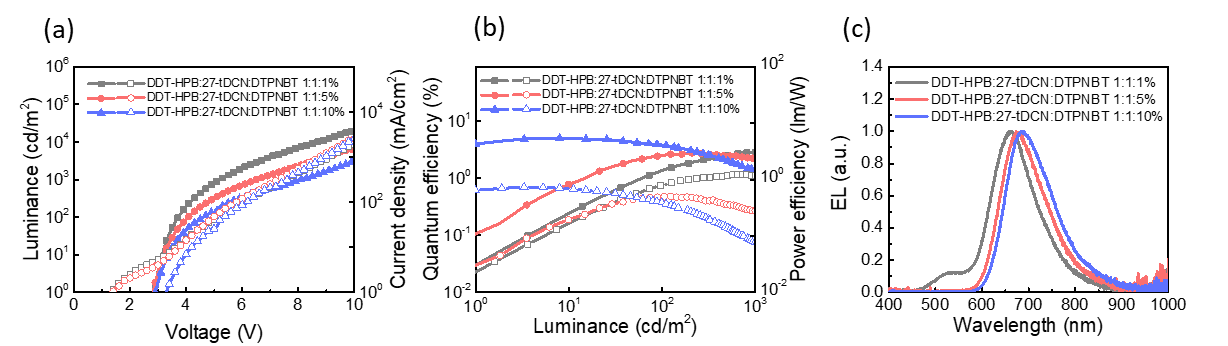


**Figure S9** different concentration of dopant DTPNBT into blend film in OLED electronic characteristic


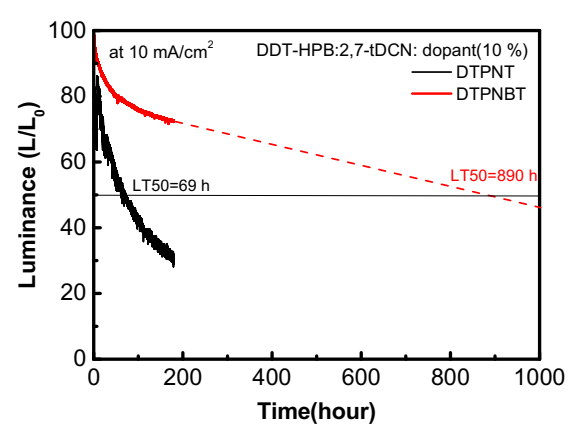


**Figure S10** Device lifetimes based on DTPNT (black line) and DTPNBT (red line) tested at current density of 10 mA/cm^2^.

**Table S1** X-ray data and structure refinement parameters for 27-DCN and 27-tDCN

| Crystal data | 27-DCN | 27-tDCN |
| --- | --- | --- |
| Empirical formula | C27 H14 N2 | C35 H32 N2 |
| Formula weight | 366.40 | 480.62 |
| Crystal system | Triclinic | Monoclinic |
| Space group | P-1 | P2**_1_**/c |
| Unit cell dimensions | a = 10.5454(8) Å a= 102.591(7)° | a = 8.3593(2) Å α= 90° |
|  | b = 10.7816(9) Å b= 92.277(6)°. | b = 24.9494(6) Å β= 94.970(2)° |
|  | c = 17.1617(13) Å g = 102.925(7)° | c = 13.1438(3) Å γ = 90° |
| Volume | 1848.0(3) Å3 | 2730.96(11) Å3 |
| Z | 4 | 4 |
| F(000) | 760 | 1024 |
| Density (calculated) | 1.317 Mg/m3 | 1.169 Mg/m3 |
| Wavelength | 0.71073 Å | 0.71073 Å |
| Cell parameters reflections used | 6883 | 7707 |
| Theta range for Cell parameters | 4.0860 to 29.8300° | 3.4250 to 29.0340° |
| Absorption coefficient | 0.078 mm-1 | 0.068 mm-1 |
| Temperature | 100(2) K | 100(2) K |
| Crystal size | 0.30 x 0.25 x 0.20 mm3 | 0.20 x 0.15 x 0.10 mm3 |
| No. of measured reflections | 18925 | 16856 |
| No. of independent reflections | 8472 [R(int) = 0.0437] | 4803 [R(int) = 0.0327] |
| Final R indices [I>2sigma(I)] | R1 = 0.0581, wR2 = 0.1516 | R1 = 0.0407, wR2 = 0.0975 |
| R indices (all data) | R1 = 0.0783, wR2 = 0.1772 | R1 = 0.0554, wR2 = 0.1085 |

**Table S2** Electroluminescence data of OLEDs based on different concentration of dopants

| EML | EL_max_  (nm) | V_on_^a^  (V) | EQE_max_^a^  (%) | CE_max_  (cd A^-1^) | PE_max_  (lm W_-1_) | at 10^3^ nits  (%) | CIE_max_  (x, y) |
| --- | --- | --- | --- | --- | --- | --- | --- |
| DDT-HPB:27-tDCN:DTPNT  1:1:1%wt | 616 | 2.4 | 5.5 | 9.23 | 12.09 | 4.2 | (0.53, 0.42) |
| DDT-HPB:27-tDCN:DTPNT  1:1:5%wt | 652 | 2.8 | 5.7 | 3.14 | 3.53 | 1.1 | (0.67, 0.32 |
| DDT-HPB:27-tDCN:DTPNT  1:1:10%wt | 660 | 2.8 | 5.8 | 2.32 | 2.61 | 0.9 | (0.68, 0.31) |
| DDT-HPB:27-tDCN:DTPNBT  1:1:1%wt | 663 | 2.7 | 3.0 | 2.04 | 1.03 | 2.9 | (0.58, 0.38) |
| DDT-HPB:27-tDCN:DTPNBT  1:1:5%wt | 673 | 2.6 | 2.8 | 0.70 | 0.48 | 2.2 | (0.696 0.31) |
| DDT-HPB:27-tDCN:DTPNBT  1:1:10%wt | 685 | 2.6 | 5.0 | 0.74 | 0.71 | 1.3 | (0.69, 0.30) |

**
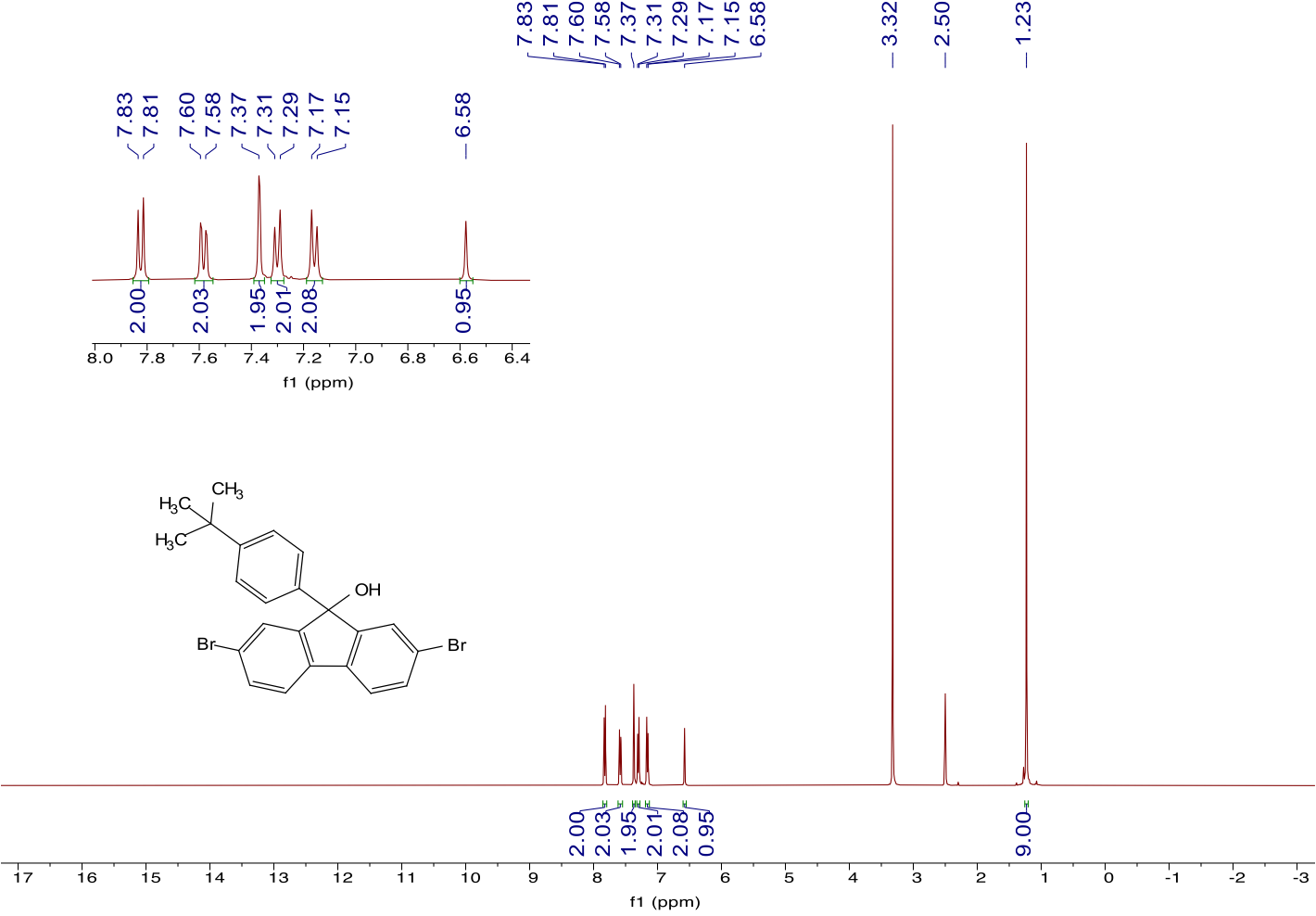
 ^1^H and ^13^C NMR spectrum**


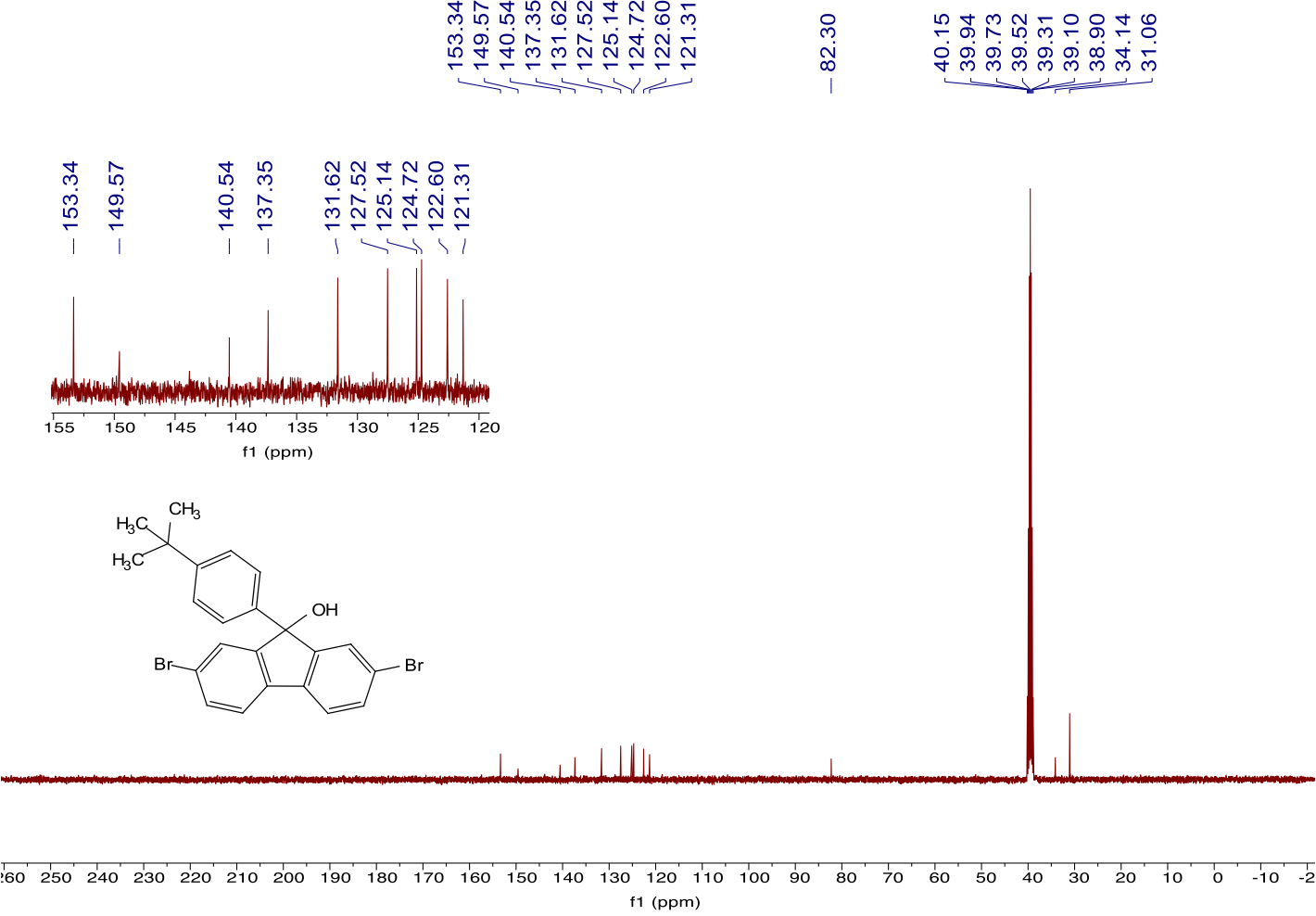


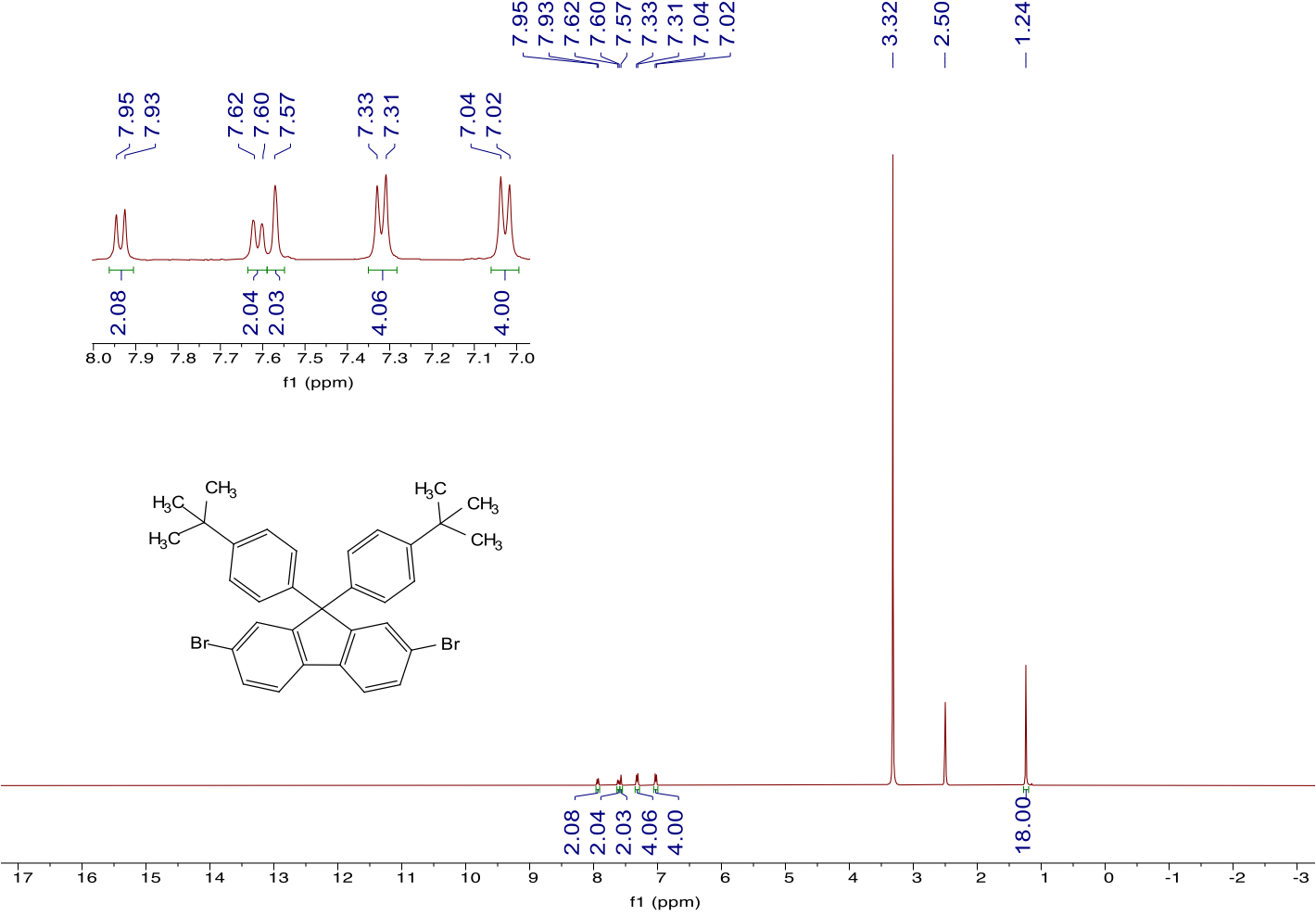

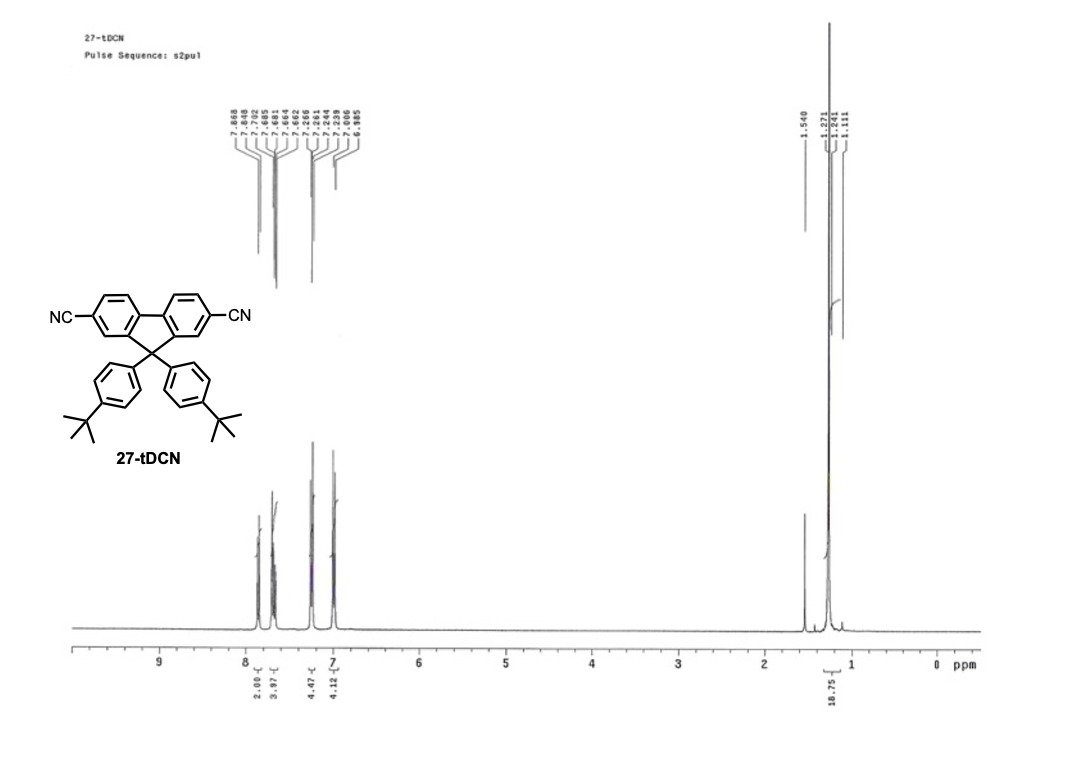


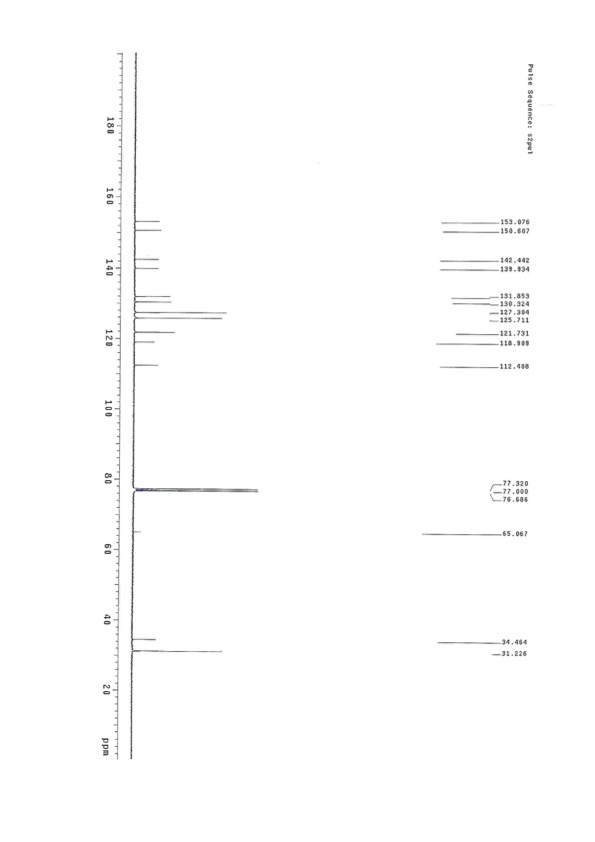

Supplement: Supplementary file 1 — Supplementary Information. [file 41598_2024_52680_MOESM1_ESM.docx]
